# COVID-induced pancreatitis: case report

**DOI:** 10.1186/s43162-021-00039-y

**Published:** 2021-03-08

**Authors:** Mohamed-Naguib Wifi, Ahmed Nabil, Abeer Awad, Reham Eltatawy

**Affiliations:** 1 Internal Medicine Department, Hepatogastroenterology Unit, Kasr Al-Ainy School of Medicine, Cairo University, Kasr Al-Aini Street, Cairo, Egypt; 2 Department of General Surgery, Kasr Al-Ainy School of Medicine, Cairo University, Cairo, Egypt

**Keywords:** COVID-19, Acute pancreatitis

## Abstract

**Background:**

Although the frequent respiratory affection in COVID-19, it is well established that it could be presented with a wide variation of gastrointestinal symptoms; however, it is the effect on the pancreas remains unclear.

**Case presentation:**

We report a case of female patient, who was diagnosed with COVID-19 infection. A week later, the patient developed an attack of acute pancreatitis. Other causes of acute pancreatitis were excluded. Therefore, this was attributed to SARS-COV2 infection.

**Conclusion:**

The case raises awareness about the possibility of acute pancreatitis in COVID-19. Also emphasize the importance of measuring serum amylase and lipase in patients with COVID-19.

## Background

From 12 December 2019 and till now, COVID-19 infection is considered a newly developed worldwide outbreak of coronavirus disease with a high rate of mortality [1] and a great impaction on health care systems worldwide [2].

Despite more frequent respiratory affection in COVID-19, it is well established now that it could present with a wide variation of gastrointestinal symptoms however it is the effect on the pancreas remains unclear [3].

The reported incidence of liver injury in COVID is up to 37%, mainly in form of abnormal ALT/AST levels; meanwhile, a pancreatic injury is reported in 17% of 54 patients in COVID Chinese infected patients [4].

Herein, we report a female patient with COVID-19 who presented with severe abdominal pain diagnosed as acute pancreatitis without any other risk factors and with non-comparable symptoms of respiratory complications.

## Case presentation

**Fig. 1:**
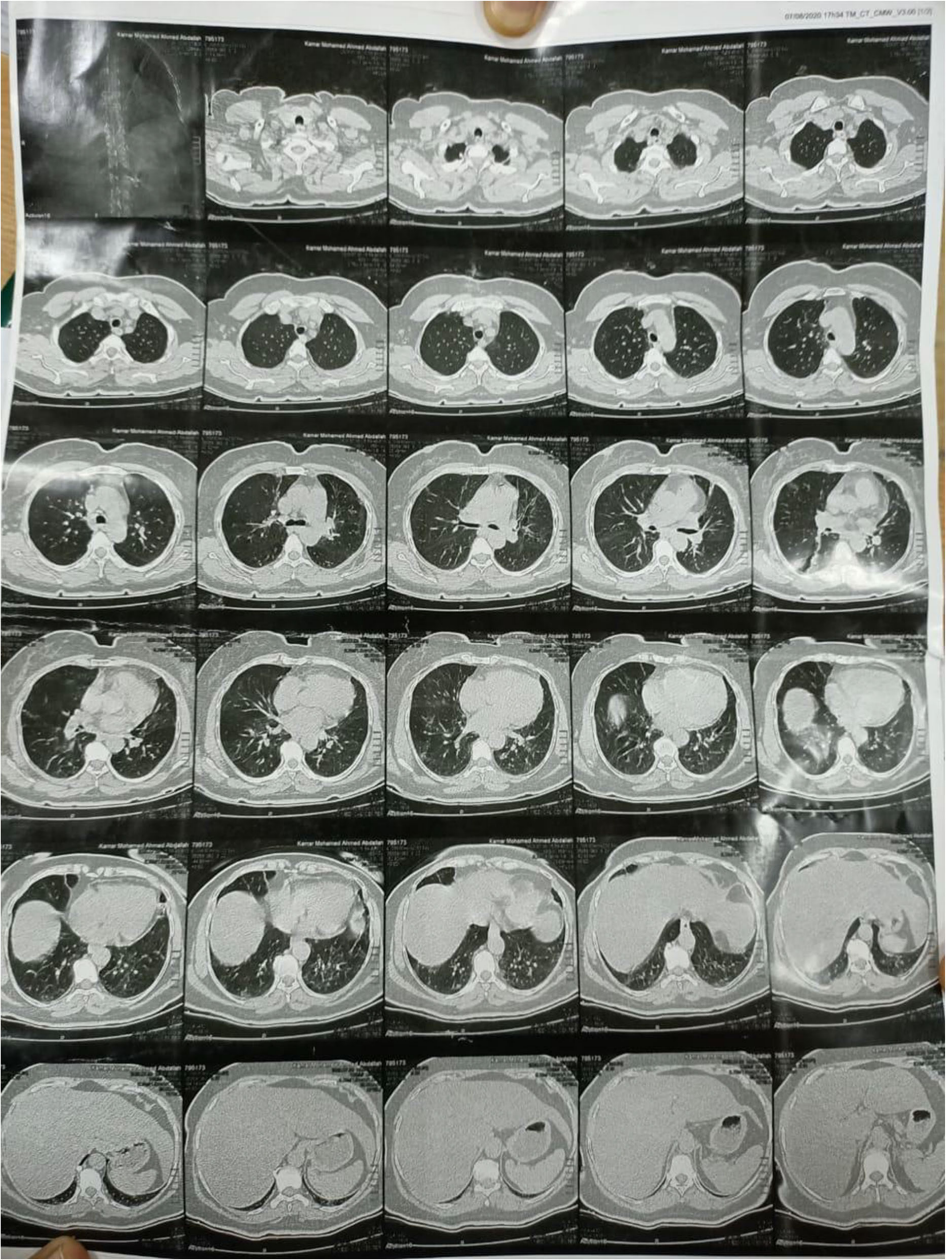
CT chest revealed mosaic attenuation with mainly subplural ground glass opacities with thickened vascular wall as well as septal thickening in keeping with small airways obstruction disease

We report a case of a 72-year-old female patient with a known medical history of hypertension and ischemic heart disease. She was on medical treatment of beta-blockers, aspirin, and nitrates. Her BMI was 33.14 kg/m^2^. The patient presented to the emergency department with an attack of severe abdominal pain associated with nausea and vomiting (7–8 times per day); the days preceded the attack of abdominal pain the patient was noticed mild cough and nasal sneezing; diagnosed as a mild COVID infection after visiting a physician in an outpatient clinic and a positive nasopharyngeal (RT-PCR) testing. The symptoms were resolved completely with home isolation and conservative treatment (paracetamol and nasal decongestants). The patient denied any history of contact with a confirmed COVID patient or a patient with flu likes symptoms. One week later, the patient complained of myalgia, anorexia, and ageusia that shortly followed by an attack of sharp diffuse colicky abdominal pain associated with nausea and vomiting (7–8 times) for which the patient presented to the emergency department for further evaluation. In the emergency room, CT chest revealed bilateral ground-glass opacities (Fig. 1). The laboratory investigations showed elevated total leucocytic count: 14.3 × 10^9^ cells/L, hemoglobin at 11.4 g/dL, CRP 118.2 mg/l, serum creatinine 1.69 mg/dL with markedly elevated serum amylase 1667 U/L and lipase: 710 U/L; other laboratory values were unremarkable (Table 1). There was no history of fever, shortness of breath, or chest pain. Vital signs showed elevated blood pressure (BP = 150/100), tachycardia (pulse100 b/min), and body temperature was normal (37.1 °C), respiratory rate of 19 breaths per minute, and her oxygen saturation measured by pulse oximetry was 95% on room air and had markedly elevated blood sugar (RBS = 500 mg/dL). On physical examination, the patient was found to have a dry mucous membrane of the tongue, pallor, and severe generalized tenderness of the abdomen with normal intestinal sounds. She also appeared fatigued and cachectic. Abdominal computed tomography was normal. The diagnosis of acute pancreatitis in a COVID-19 patient was settled. Upon that the patient was referred to the ICU room in the quarantine hospital, 3 days at ICU, the condition was resolved with conservative treatment, bowel rest, intravenous fluids, and analgesia; the patient was discharged after 15 days of admission. Her BMI on discharge was 25.25 kg/m^2^; she lost 20 kg from her weight and her blood sugar returned to the normal level.

**Table 1:** Laboratory results on admission

Laboratory test	Level	Normal range
WBC count (109 cells/L)	14.3 × 10^9^ cells/L	3.5–9.5 10^9^ cells/L
Neutrophil (%)	75%	50–70%
Lymphocyte (%)	20%	20–40%
RBC count (109 cells/L)	6.01	3.9–5.6 10^9^ cells/L
Hemoglobin (g/dL)	11.4	12–16 g/dL
Platelet count (10^9^ cells/L)	464	125–350 10^9^ cells/L
Hematocrit (%)	44.1	36−48%
AST (U/L)	33	5–45 U/L
ALT (U/L)	20	5–40 U/L
Albumin (g/L)	4	3.5–5.5 mg/L
Amylase (U/L)	1667	0–140 U/L
Lipase (U/L)	710	0–60 U/L
Blood sodium level (mEq/L)	135	135–145 mEq/L
Blood potassium level (mEq/L)	3.7	3.6–5.2 mEq/L
Blood calcium level (mg/dL)	8.6	8.5−10.5 mg/dL
Triglyceride (mg/dL)	81	40–150 mg/dL
Blood urea nitrogen (mg/dL)	55	8–20 mg/dL
Creatinine (mg/dL)	1.69	0.5–1.2 mg/dL

## Conclusion

The most common prevalent symptoms of COVID-19 infection are fever, cough, and dyspnea; however, there are a wide range of symptoms that are reported frequently with this infection as gastrointestinal symptoms of nausea, vomiting, and diarrhea [5].

A study in China reported elevated lipase, in up to 17% of active COVID-19 cases and that the incidence of pancreatic injury is not so low in the COVID-19 pneumonia patients [4].

In COVID, pancreatic injury could be explained by the expression of Angiotensin-converting enzyme-2 (ACE-2) receptors on pancreas [6] with subsequent injury to the islet of the pancreas with an elevation of serum amylase and lipase enzymes and risk of development of acute diabetes as in our case. However, some authors attributed pancreatic injury as a part of systemic inflammatory response in COVID-19 pneumonia [4].

A retrospective cohort study across 6 US centers concluded that about 48% of patients with elevated lipase more than 3 times the upper limit of normal were due to non-pancreatic etiologies, as gastritis/gastroparesis [7]. Fortunately, no patient developed acute pancreatitis [3]. Meanwhile, Hadi et al. reported severe acute pancreatitis in two first degree relatives with COVID [8].

Other cohorts’ studies reported that up to 16% of patients with COVID from approximately 40% of presenting with gastrointestinal symptoms developed elevated serum amylase and lipase, with only 7% had significant pancreatic changes on CT [9].

Normal abdominal imaging could be explained by mild pancreatic injury induced by the SARS-COV-2 virus. Awareness to the less common presentation of COVID-19 infection is crucial to avoid the misdiagnosis and delay in the proper management.

In summary, there is a need to raise the suspicion of COVID infection in unexplained abdominal pain that has a potential role in the reduction of transmission to other patients and hospital staff.

Also, further researches are warranted to evaluate whether clinical pancreatitis considers one of COVID presentation, concomitant disease entity, or a subsequent complication.

## Data Availability

Not applicable

## Abbreviations

SARS-COV2: Severe acute respiratory syndrome coronavirus 2
ALT: Alanine transaminase
AST: Aspartate aminotransferase
RT-PCR: Real-time polymerase chain reaction
CT: Computerized tomography
ICU: Intensive care unit
ACE-2: Angiotensin-converting enzyme-2

## References

[1] Wu F, Zhao S, Yu B (2020). A new coronavirus associated with human respiratory disease in China. Nature..

[2] Oba A, Stoop TF, Löhr M, et al (2020) Global Survey on Pancreatic Surgery During the COVID-19 Pandemic. Ann Surg 272(2):e87-93. 10.1097/SLA.0000000000004006. 10.1097/SLA.0000000000004006 PMC7268883 32675507

[3] McNabb-Baltar J, Jin DX, Grover AS (2020). Lipase elevation in patients with COVID-19. Am J Gastroenterol.

[4] Wang F, Wang H, Fan J, Zhang Y, Wang H, Zhao Q (2020) Pancreatic injury patterns in patients with COVID-19 pneumonia [published online ahead of print, 2020 Apr 1]. Gastroenterology S0016-5085(20)30409-1. 10.1053/j.gastro.2020.03.055 10.1053/j.gastro.2020.03.055 PMC7118654 32247022

[5] Guan WJ, Ni ZY, Hu Y, et al (2020) Clinical Characteristics of Coronavirus Disease 2019 in China. N Engl J Med 382(18):1708-20. 10.1056/NEJMoa2002032. 10.1056/NEJMoa2002032 PMC7092819 32109013

[6] Liu F, Long X, Zhang B, Zhang W, Chen X, Zhang Z (2020). ACE2 expression in pancreas may cause pancreatic damage after SARS-CoV-2 infection. Clin Gastroenterol Hepatol.

[7] Jin DX, Yang AL, Suleiman SL (2019). Marked serum lipase elevations are associated with longer hospitalizations in patients with non-pancreatic hyperlipasemia. Gastroenterology.

[8] Hadi A, Werge M, Kristiansen KT (2020). Coronavirus disease-19 (COVID-19) associated with severe acute pancreatitis: case report on three family members. Pancreatology.

[9] Mukherjee R, Smith A, Sutton R (2020). Covid-19-related pancreatic injury. Br J Surg.

